# Primary prevention of myocardial infarction with angiotensin-converting enzyme inhibitors and angiotensin receptor blockers in hypertensive patients with rheumatoid arthritis—A nationwide cohort study

**DOI:** 10.1371/journal.pone.0188720

**Published:** 2017-12-07

**Authors:** Ting-Tse Lin, Cho-Kai Wu, Min-Tsun Liao, Yao-Hsu Yang, Pau-Chung Chen, Dong-Feng Yeih, Lian-Yu Lin

**Affiliations:** 1 Department of Internal Medicine, National Taiwan University Hospital Hsin-Chu Branch, Hsin-Chu, Taiwan; 2 College of Medicine, National Taiwan University, Taipei, Taiwan; 3 Institute of Biomedical Engineering, National Chiao-Tung University, Hsinchu, Taiwan; 4 Division of Cardiology, Department of Internal Medicine, National Taiwan University College of Medicine and Hospital, Taipei, Taiwan; 5 Department of Traditional Chinese Medicine, Chiayi Chang Gung Memorial Hospital, Chiayi, Taiwan; 6 Health Information and Epidemiology Laboratory of Chang Gung Memorial Hospital, Chiayi, Taiwan; 7 School of Traditional Chinese Medicine, College of Medicine, Chang Gung University, Taoyuan, Taiwan; 8 Institute of Occupational Medicine and Industrial Hygiene, National Taiwan University College of Public Health, Taipei, Taiwan; 9 Department of Internal Medicine, Fu-Jen Catholic University Hospital, New Taipei City, Taiwan; 10 Department of Internal Medicine, School of Medicine, Fu-Jen Catholic University, New Taipei City, Taiwan; Cleveland Clinic Lerner Research Institute, UNITED STATES

## Abstract

**Background:**

Rheumatoid arthritis (RA) is regarded as a high risk factor for myocardial infarction. Hypertension is a major modifiable risk factor contributing to increased risk of myocardial infarction (MI). Dual blood pressure (BP)-lowering and anti-inflammatory effect of renin-angiotensin-system (RAS) inhibitors may possess protective effect from MI in RA population. However, treatment of hypertension with RAS inhibitors and MI in RA population remains unclear.

**Methods:**

We investigated whether RAS blockade could decrease risk of incident MI in hypertensive patients with RA. We identified patients with RA and hypertension from the Registry for Catastrophic Illness, a nation-wide database encompassing almost all of the RA patients in Taiwan from 1995 to 2008. The primary endpoint was MI and the median duration of follow up was 2,986 days. Propensity score weighting and Cox proportional hazards regression models were used to estimate hazard ratios for MI.

**Results:**

Among 27,335 subjects, 9.9% received angiotensin-converting enzyme inhibitors (ACEIs), 25.9% received angiotensin II receptor blockers (ARBs) and 20.0% received ACEIs or ARBs alternatively. The incidence of MI significantly decreased in patients treated with ACEIs (hazard ratio 0.707; 95% confidence interval 0.595–0.840), ARBs (0.641; 0.550–0.747) and ACEIs/ARBs (0.631; 0.539–0.739). The protective effect of ACEI or ARB therapy was significantly better in patients taking longer duration. The effect remained robust in subgroup analyses.

**Conclusions:**

Therapy of ACEIs or ARBs is associated with a lower risk of MI among patients with RA. Hence, hypertension in patients with RA could comprise a compelling indication for RAS inhibitors.

## Introduction

Rheumatoid arthritis (RA) is a common autoimmune disease characterized by chronic synovial inflammation and is associated with progressive disability, systemic complications, and early death[1]. The risk of sudden cardiac death and ischemic heart disease (IHD) is significantly higher in RA than it is in non-RA subjects, largely contributing to RA mortality[2, 3]. The increased rates are not explained by traditional risk factors [4] but strongly associated with systemic inflammation and disease activity markers[5]. Besides, several studies have revealed the relevance of a genetic component in the development of cardiovascular disease in RA patients[6]. Furthermore, RA related inflammatory cytokines (interleukin-6 and TNF-α), acute-phase reactants and immune complexes have been proved to increase endothelial activation and atheromatous plaque vulnerability[7]. Thereby, among patients with RA, responders to anti-TNFα biologic therapies could markedly reduce the risk of myocardial infarction (MI) when compared to non-responders[8].

The administration of angiotensin converting enzyme inhibitors (ACEIs) and angiotensin II receptor blockers (ARBs) improves cardiac function and reduces mortality in post-MI patients[9]. In addition to blood pressure (BP) lowering, the protective effect of renin-angiotensin-system (RAS) blockade might come from attenuation of ventricular remodeling [10]; decrease in sympathetic activity[11], and improvement of endothelium function and plaque stabilization[12]. RAS blockade was also independently associated with enhanced the function of islet beta cells in RA patients with high-grade inflammation[13]. As mentioned above, systemic inflammation could accelerate coronary atherosclerosis and result in higher prevalence of IHD in RA. The pleiotropic effects of RAS inhibitors could be expected to reduce the incidence of IHD and MI.

In the general population, ACEIs and ARBs have been proved to reduce cardiovascular (CV) mortality, especially in high-risk subjects [12, 14]. Regarding to CV disease management in RA population, the European league against rheumatism (EULAR) recognized RA as a high CV risk and hypertension as a major modifiable risk factor contributing to increased risk of CV events [15]. The 2010 EULAR guideline for cardiovascular management in RA patients recommended ACEIs and ARBs as preferred treatment options in those with hypertension [16]. However, this recommendation has been omitted in the 2017 EULAR guideline [15] since only various small randomized control studies supporting this recommendation [17, 18]. To fill the gap, we hypothesize that the use of ACEI or ARB is associated with risk reduction of MI in RA patients with hypertension in a nationwide cohort.

## Materials and methods

### Data source

We used integrated medical and pharmacy claims data from National Health Insurance Research Database (NHIRD) in Taiwan. Since 1995, the NHIRD is an administrative claims dataset which captures 99% of all medical claims for the Taiwanese citizens. When patients are diagnosed as RA, they are registered in the ‘Catastrophic Illnesses’ system according to our NHI policy. Upon registering in the system, the loss to follow rate could substantially be reduced since almost all the medical fees could be waived. All the medications, procedures, every outpatient clinic visits, and hospital admission covered by the insurance were recorded in the database. In addition, to validate the diagnosis in the database, the Bureau of National Health Insurance routinely reviewed the original medical charts of all of the patients who applied for catastrophic illness registration. To comply with data privacy regulations, personal identities were encrypted, and all data were analyzed in a de-identified manner (S1 File). The protocol for this study was approved by the Institutional Review Board of National Taiwan University Hospital.

### Study population

**Fig 1:**
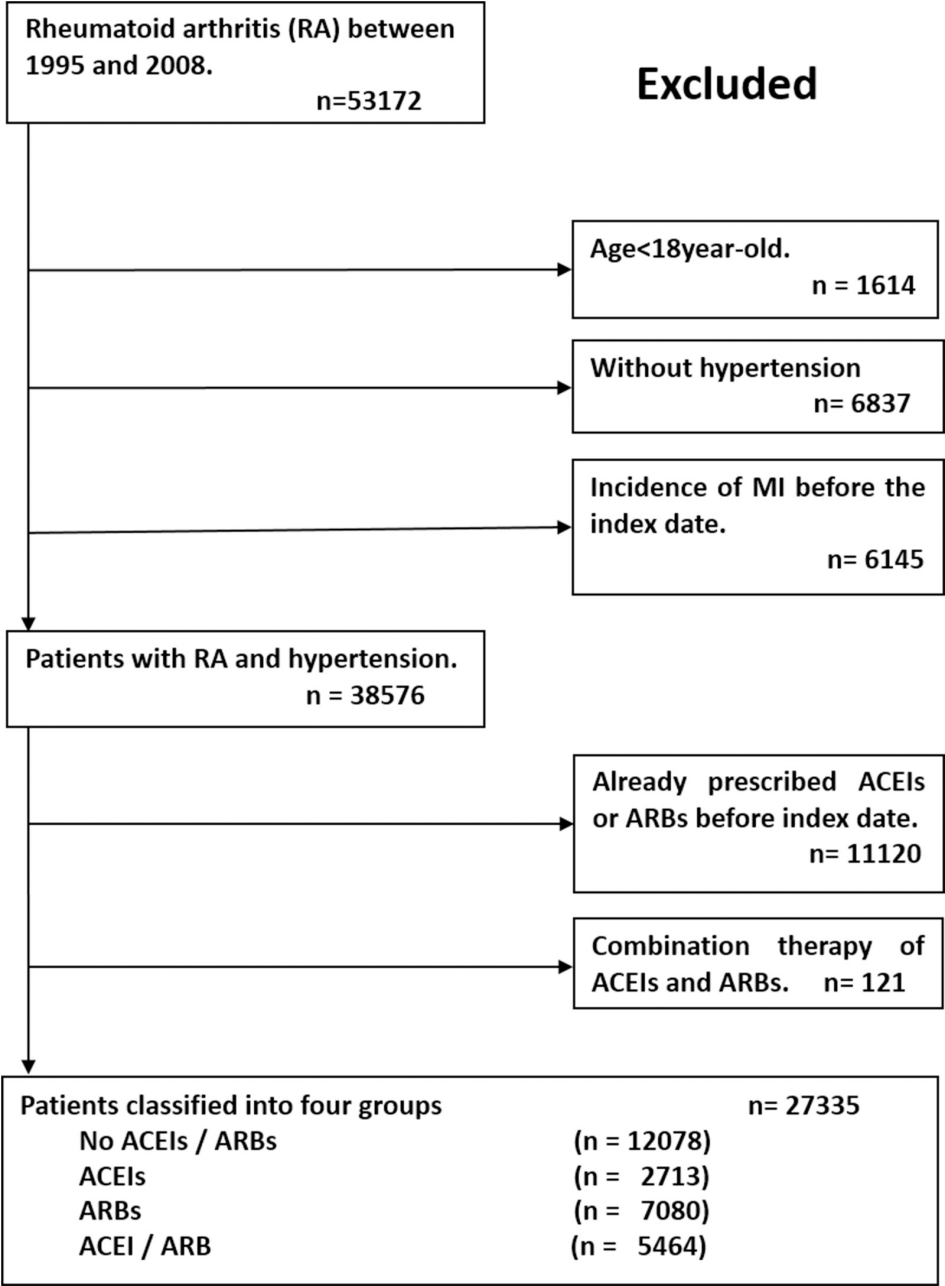
Patient flow diagram. The index date was identified as the date of the first time diagnosis of RA. Abbreviations: ACEIs, angiotensin converting enzyme inhibitors; ARB, angiotensin receptor blockers; MI, myocardial infarction.

We identified RA subjects through the use of International Classification of Disease, Ninth Revision, Clinical Modification (ICD-9-CM) code 714.0 to 714.9 without juvenile chronic polyarthritis (714.3) in the catastrophic illness file from 1995 to 2008. A flowchart of the process used to identify study subjects is presented in Fig 1. The cohort from 1995–2008 of RA patients was 53172 subjects. We excluded patients younger than 18 years (n = 1614), without diagnosis of hypertension (n = 6837) and incident MI before the diagnosis of RA (index date) (n = 6145). The identification of hypertension was based on ICD9-CM codes in two consecutive ambulatory visit or discharge diagnoses. The index date for the study cohort was identified as the date of the first time diagnosis of RA. In order to investigate the protective effect of ACEIs and ARBs, we excluded subjects who previously took ACEIs or ARBs before the index date (N = 11120) and those who received combination therapy of ACEIs and ARBs (n = 121). The final enrolled cohort was 27335 subjects. Subsequently, we divided them into four groups including subjects without taking ACEIs or ARBs (n = 12078), subjects received ACEIs only (n = 2713), subjects received ARBs only (n = 7080) and finally subjects received ACEIs or ARBs alternatively (n = 5364).

### Drug use, covariates and outcome

The criteria to define the ACEIs or ARBs users were taking these medications for more than 28 days during the following period. In Taiwan, we had 10 types of ACEIs (around 90 of generic drugs with different doses) and 7 types of ARBs (around 60 of generic drugs). The majority of the treatment frequencies for ACEIs and ARBs were once daily (qd; around 60% and 80% respectively) and twice a day (bid; around 30% and 20% respectively). In the current study, the total treatment duration of ACEIs, ARBs, or ACEIs/ARBs was utilized to demonstrate the time–response effect[19]. In terms of comorbidities, diabetes, dyslipidemia, ischemic stroke, hemorrhagic stroke, coronary artery disease, heart failure hospitalization, or peripheral artery disease were recorded within the last 12 months prior to the index date. Medications including anti-platelet, beta-blockers, calcium channel blockers (CCB), statin, disease modifying anti-rheumatic drugs (DMARD), steroid, non-steroid anti-inflammation drugs (NSAIDs), oral antidiabetic drugs (OADs) and insulin, were identified.

The aim of this study was to investigate the incidence of MI between RAS blockade users and non-user during long-term follow-up. The clinical outcome was MI, identified by using ICD-9-CM codes for STEMI and NSTEMI in any ambulatory visit and discharge diagnoses. The corresponding ICD-9-CM codes for comorbidities and outcomes were listed in S1 Table.

### Statistical analyses

We used one-way ANOVA for continuous variables and chi-square test for categorical variables to compare the baseline characteristics among four groups. Owing to the heterogeneity of four groups, we utilized multivariate Cox’s proportional hazard regression analyses to derive the adjusted hazard ratios (HRs) for incidence of MI in different groups. The model was adjusted for all confounders (age, gender, comorbidities, and medication usage) as main results. Furthermore, to balance the differences among four groups, the propensity scores were constructed using multinomial logistic regression to model the receipt of ACEIs or ARBs as a function of baseline patient characteristics[20]. The covariates in this multinomial logistic regression model included all the background characteristics listed in Table 1 including age, gender, comorbidities, and medications. The propensity score-based adjustment was conducted to remove the initial bias. The procedure included combination of propensity scores with other covariates in a regression model, the inverse probability of treatment weights (IPTW) and marginal mean weighting through stratification (MMWS)[19, 21, 22]. In order to avoid profound reductions in the study population leading to lack of generalizability of the findings, propensity score matching was avoided. We used the Kaplan–Meier method to illustrate the event-free survival curves of the four groups. The log-rank test was applied to test the differences in survival among groups. We conducted subgroup analyses stratified by age, gender, diabetes, hypertension, use of DMARD and steroid. To deal with the discontinuation of ACEIs or ARBs over time, we performed sensitivity analyses with follow-up time truncated at 2 years, 5 years and 8 years.

**Table 1:** Demographic and clinical characteristics of study subjects.

	Control	ACEIs	ARBs	ACEIs/ARBs
n (%)	12078 (44.2)	2713 (9.9)	7080 (25.9)	5464 (20.0)
Age (mean)	51	60*	59*	60*
18–64 (%)	82.3	62.3*	67.7*^,^^†^	67.1*^,^^†^
65–74 (%)	12.3	25.1*	23.3*^,^^†^	25.1*^,†,^^‡^
≧75 (%)	5.5	12.6*	9.9*^,^^†^	7.7*^,^^†^^,^^‡^
Gender, female %	78.3	73.9*	77.9^†^	79.4*^,^^†^^,^^‡^
Diabetes, %	10.7	30.2*	28.9*	39.6*^,^^†^^,^^‡^
Dyslipidemia	29.5	34.5*	42.9*^,^^†^	47.7*^,^^†^^,^^‡^
Ischaemic stroke/TIA, %	2.6	10.6*	7.6*^,^^†^	13.1*^,^^†^^,^^‡^
Haemorrhagic stroke, %	0.7	2.3*	2.1*	3.3*^,^^†^^,^^‡^
CAD, %	21.4	32.2*	34.6*^,^^†^	44.4*^,^^†^^,^^‡^
PAD, %	15.0	23.9*	21.6*^,^^†^	27.4*^,^^†^^,^^‡^
Heart failure hospitalization, %	10.6	18.0*	12.2*^,^^†^	22.4*^,^^†^^,^^‡^
Medications				
Anti-platelet	19.8	27.9*	29.1*^,^^†^	45.0*^,^^†^^,^^‡^
Beta-blockers	38.6	55.7*	57.1*	77.6*^,^^†^^,^^‡^
CCBs	55.1	66.4*	75.6*^,^^†^	88.8*^,^^†^^,^^‡^
Statin	20.4	22.0*	30.0*^,^^†^	38.4*^,^^†^^,^^‡^
DMARD	48.7	57.3*	59.6*^,^^†^	64.7*^,^^†^^,^^‡^
NSAIDs	71.2	77.0*	78.6*^,^^†^	79.3*^,^^†^^,^^‡^
Steroid	70.8	79.4*	81.9*^,^^†^	87.1*^,^^†^^,^^‡^
OADs	10.4	22.9*	22.0*	33.4*^,^^†^^,^^‡^
Insulin	2.5	7.0*	6.0*^,^^†^	12.0*^,^^†^^,^^‡^

* p<0.05 when compared with control

**†** p<0.05 when compared with ACEIs group

^**‡**^ p<0.05 when compared with ARBs group

Abbreviations: ACEIs, angiotensin converting enzyme inhibitors; ARBs, angiotensin receptor blockers, CAD, coronary artery disease; CCBs, calcium channel blockers, DMARD, disease modifying anti-rheumatic drugs;; NSAIDs, non-steroid anti-inflammation drugs; OADs, oral anti-diabetic drugs; PAD, peripheral artery disease

For all HRs, we calculated 95% CIs. All p values were two-sided and a p value <0.05 was considered statistically significant. All of the analyses were performed using the Statistical Package for the Social Sciences (SPSS) for Windows, Version 19.0 (SPSS, Inc., Chicago, IL).

## Results

### Patient characteristics

There were 27,335 patients enrolled in our cohort. Among them, 12,078 (44.2%) did not use ACEIs or ARBs, while 2,713 (9.9%) used ACEIs, 7,080 (25.9%) used ARBs, and 5,464 (20.0%) used ACEIs or ARBs alternatively (ACEIs/ARBs). In the ACEIs or ARBs alternative treatment group, only less than two percent of patients received simultaneous ACEIs and ARBs treatment. The algorithm is listed in Fig 1.

The median of follow-up time was 2,986 days. Subjects’ clinical and demographic characteristics are listed in Table 1. Subjects without taking ACEIs or ARBs were served as control group. As demonstrated in Table 1, subjects not on ACEIs or ARBs were significantly younger than that in other three groups and there were fewer female patients in the ACEIs group as compared with other groups. The prevalence of cardiovascular risk factors (diabetes and dyslipidemia) were significantly higher in ACEIs, ARBs and ACEIs/ARBs group as compared with control group. Likewise, the comorbidities (stroke, coronary artery disease, peripheral arterial disease and heart failure) in these three groups were more common than that in control group. In regard to medications, as compared with control group, anti-platelet agents (27.9%, 29.1% vs. 19.8%), beta-blockers (55.7%, 57.1% vs. 38.6%), calcium channel blockers (66.4%, 75.6% vs. 55.1%), statin (22.0%, 30.0% vs. 20.4%), DMARD (57.3%, 59.6% vs. 48.7%), NSAIDs (77.0%, 78.6% vs. 71.2%), steroid (79.4%, 81.9% vs. 70.8%), OADs (22.9%, 22.0% vs. 10.4%) and insulin (7.0%, 6.0% vs. 2.5%) were more frequently prescribed in the ACEIs and the ARBs groups. Finally, all the medications were more frequently prescribed in the ACEIs/ARBs group than that in other three groups.

### Main outcome: MI

The crude incidence rate of MI was 6.0% during follow-up period. Compared with control group (6.2%), the incidence was higher in subjects receiving ACEIs (7.4%), and ACEIs/ARBs (7.0%) but lower in subjects receiving ARBs (4.3%) (Table 2). However, after transforming the incidence into patient-years, the incidence was lower in RAS blockade groups (ACEIs, 7.2 per 1000 patient-years; ARBs, 4.7 per 1000 patient-years; ACEIs/ARBs, 5.9 per 1000 patient-years) than control groups (7.6 per 1000 patient-years). This might imply that patients without RAS inhibitors were at increased risk of CV disease in our cohort.

**Table 2:** Incidence of acute myocardial infarction by prescriptions.

	Incidence of AMI				
	Total	Control	ACEIs	ARBs	ACEIs/ARBs
Number of patients	27335	12078	2713	7080	5464
Duration of follow-up Median (IQR), days	2986 (1484,4401)	2554 (1175,4439)	3562 (2177,5275)	3129 (1730,4909)	4168 (3050,5704)
Mean (SD), days	3005 (2038)	2840 (2035)	3578 (1942)	3271(1966)	4103(1734)
Incident cases—n (%)	1640 (6.0)	748 (6.2)	202 (7.4)	306 (4.3)	384 (7.0)
Incidence per 1000 patient-years	6.2	7.6	7.2	4.7	5.9

Abbreviations

AMI, acute myocardial infarction; ACEIs, angiotensin converting enzyme inhibitors; ARB, angiotensin receptor blockers; IQR, interquartile range; SD, standard deviation

After adjusting for potential confounders, by using control group as reference, treatment with ACEIs (adjusted HR, 0.707; 95% confidence interval (CI): 0.595–0.840), ARBs (adjusted HR, 0.641; 95% CI: 0.550–0.747) and ACEIs/ARBs (adjusted HR, 0.631; 95% CI: 0.539–0.739) were associated with lower risk of developing MI (Table 3). The results remain similar after adding propensity scores in the Cox regression model. The adjusted HR for MI was 0.716 (95% CI: 0.603–0.851), 0.652 (95% CI: 0.559–0.760) and 0.633 (95% CI: 0.540–0.742) associated with ACEIs, ARBs and ACEIs/ARBs, respectively. Interaction analyses showed that the HR was significantly higher in the ACEIs than in the other two groups.

**Table 3:** Hazard ratios (95% CI) of developing acute myocardial infarction in patients taking ACEI, ARB, or ACEI/ARB, with no RAS blockade treatment as the control group.

	ACEIs versus control	ARBs versus control	ACEIs/ARBs versus control	P-value
Overall, HR (95% CI)				
Adjusted HR	0.707 (0.595–0.840)	0.641 (0.550–0.747)	0.631 (0.539–0.739)	<0.001
Adjusted HR-PS adjustment	0.716 (0.603–0.851)	0.652 (0.559–0.760)	0.633 (0.540–0.742)	<0.001
*Period of treatment ≦ 180 days*				
Adjusted HR	0.798 (0.640–0.972)	0.728 (0.621–0.943)	0.979 (0.663–1.444)	0.015
Adjusted HR- PS adjustment	0.802 (0.762–0.964)	0.807 (0.712–0.912)	0.994 (0.673–1.468)	0.025
*Period of treatment 180–360 days*				
Adjusted HR	0.760 (0.608–0.949)	0.733 (0.578–0.930)	0.829 (0.701–0.951)	0.012
Adjusted HR- PS adjustment	0.774 (0.619–0.968)	0.744 (0.586–0.945)	0.823 (0.699–0.941)	0.038
*Period of treatment ≧ 360 days*				
Adjusted HR	0.662 (0.506–0.866)	0.559 (0.462–0.675)	0.578 (0.484–0.689)	<0.001
Adjusted HR- PS adjustment	0.652 (0.487–0.812)	0.570 (0.471–0.689)	0.575 (0.481–0.688)	<0.001
P for time trend	<0.05	<0.05	<0.05	

Abbreviations

ACEIs, angiotensin converting enzyme inhibitors; ARB, angiotensin receptor blockers; CI, confidence interval; HR, hazard ratio

We stratified the period of treatment into three categories which were ≦ 180 days, 180 to 360 days and ≧ 360 days. For patients treated with ACEIs, the adjusted HR was 0.798 (95% CI: 0.640–0.972), 0.760 (95% CI: 0.608–0.949) and 0.662 (95% CI: 0.506–0.866) respectively. For ARBs, the adjusted HR was 0.728 (95% CI: 0.621–0.943), 0.733 (95% CI: 0.578–0.930) and 0.559 (95% CI: 0.462–0.675) respectively. For ACEIs/ARBs, the adjusted HR was 0.979 (95% CI: 0.663–1.444), 0.829 (95% CI: 0.701–0.951) and 0.578 (95% CI: 0.484–0.689) respectively. There was a time–response relationship for the period of treatment and the prevention of MI (P<0.05). Notably, the HR did not reach significance in alternative ACEIs and ARBs group when the treatment duration was less than 180 days. The statistical results of other two propensity score-based models, IPTW and MMWS, are listed in S2 Table. The sensitivity analysis was performed in accordance with follow-up period truncated at 2 years, 5 years and 8 years. The result was shown in S3 Table.

**Fig 2:**
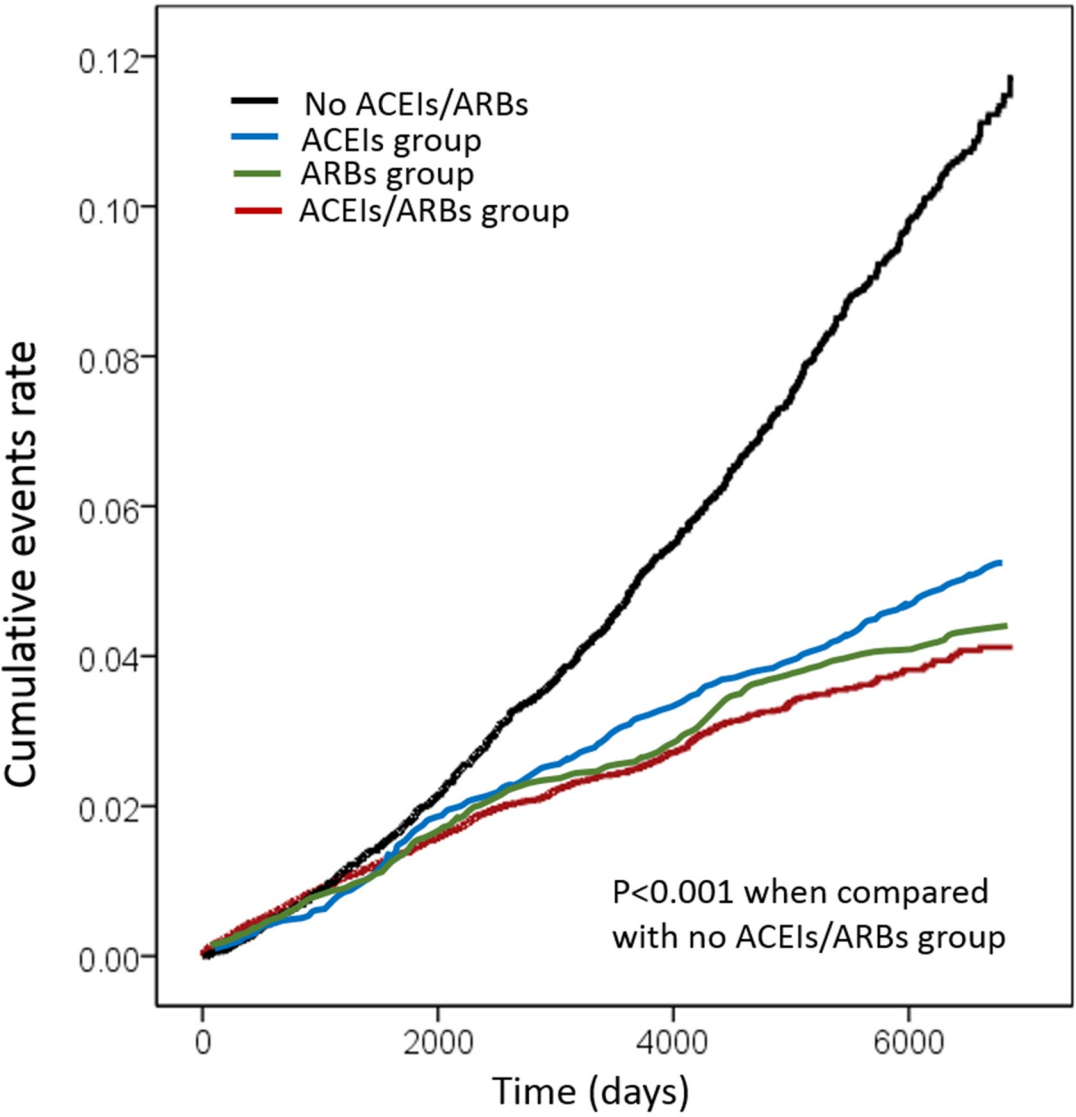
Kaplan–Meier curves showing the incidence of myocardial infarction among patients with ACEI (blue), ARB (green) or ACEI/ARB (red) treatment and controls (black). The log-rank analysis showed significant different (P < 0.001).

**Fig 3:**
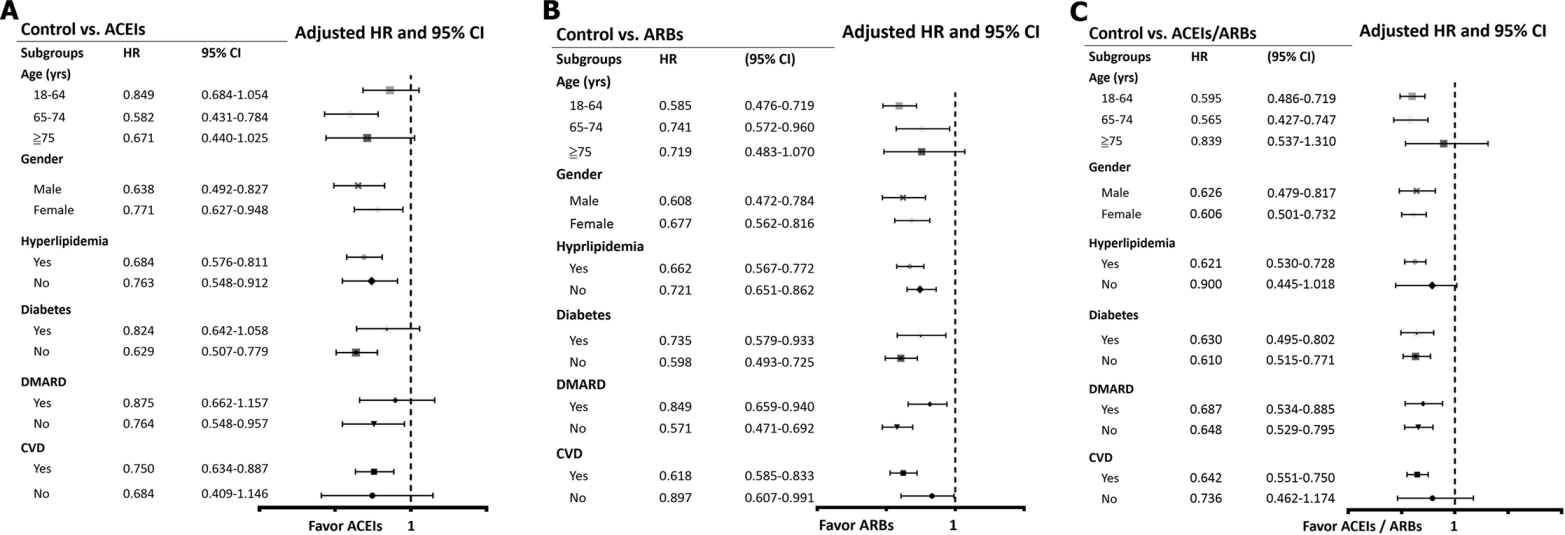
Subgroup analyses. (A) Hazard ratios of myocardial infarction in specific subgroups of ACEIs treated patients by using controls as reference group. (B) Hazard ratios of myocardial infarction in specific subgroups of ARBs treated patients by using controls as reference group. (C) Hazard ratios of myocardial infarction in specific subgroups of ACEIs/ARBs treated patients by using controls as reference group. Abbreviations: ACEIs, angiotensin converting enzyme inhibitors; ARB, angiotensin receptor blockers; HR, hazard ratio; CI, confidence interval; CVD, cardiovascular disease (combination of coronary artery disease, peripheral artery disease and hospitalization for heart failure); DMARD, disease modifying anti-rheumatic drugs.

The Kaplan–Meier survival curves are illustrated in Fig 2. The log-rank test was significant in the RAS blockades vs. control group (P<0.001). Subgroup analyses demonstrated that the RAS blockade treatment groups are associated with better outcome in most subgroups (Fig 3). Nevertheless, for patients treated with ACEIs, there was no obvious protective effect from MI in subgroups with age of 18 to 64 and older than 75 years, with diabetes, with DMARD treatment and without cardiovascular disease (CVD). For ARBs, patients with age ≧ 75 years was not associated with reduced risk of MI. Similarly, in the ACEIs/ARBs group, patients with age ≧ 75 years and without CVD did not achieve significant risk reduction of MI. At last, the results still remained similar when comparing the baseline characteristics and HR of developing AMI between non-user (control group) and user of renin-angiotensin-system inhibitors (ACEI+ARB+ACEI/ARB group). The data was listed in S4 and S5 Tables.

## Discussion

In this nationwide large cohort study, we found that RAS inhibitors was associated with around 30–40% lower risk of developing MI among patients with RA. There was a “dose-response” relationship between treatment duration and the degree of MI risk reduction. The results remain robust in most subgroups. The protective effect of RAS inhibitors might be through anti-inflammatory and BP-lowering effects in the way that diabetic patients share[23]. To our knowledge, our study is the first to evaluate the association of RAS inhibitors with MI in RA patients with hypertension.

As we know, the prevalence of hypertension in RA patients is as high as 70%[24]. On the other hand, 31% to 59% patients with MI have antecedent hypertension[25]. Hypertension is also a leading risk factor for ischemic heart disease and coronary interventions[26]. A meta-analysis has demonstrated that every 10 mm Hg reduction in systolic blood pressure is associated with a 27% reduction of MI in general population apart from which kinds of anti-hypertensive drugs were used[27]. However, the mechanisms through which hypertension contributes to the occurrence of myocardial infarction should focus on RAS[28]. Treatment of RAS inhibitors exerts particular benefits and is compelling to lower blood pressure in that high-risk hypertensive subgroups, including patients with diabetes, chronic kidney disease and heart failure[29]. Given RA recognized a higher risk for further cardiovascular events, RAS blockade might probably be considered as first-line therapy in RA patients with hypertension. Results of our study support the treatment preferences which has been recommended in the prior guideline[16].

Systemic release of pro-inflammatory cytokines from RA synovial tissue could boost the immune-inflammatory process, directly accelerating atherosclerosis, increasing plaque vulnerability, and indirectly promoting insulin resistance, endothelium dysfunction, dyslipidemia and prothrombosis[30, 31]. The adverse effects both on vascular injury and risk factors promotion synergistically contribute to worse outcomes in RA. Anti-inflammatory therapy with TNF-inhibitor or methotrexate not only control the disease activity but also significantly reduce risks of major CV events in patients with RA[8, 32]. Differing from the mechanism of anti-rheumatic drugs, ACEIs and ARBs could antagonize the actions of angiotensin II, a peptide that plays significant role in the pathogenesis of hypertension, renal disease and CVD by exerting a pro-inflammatory effect via angiotensin II type 1 receptor (AT1R)[33]. Activation of AT1R both locally and systemically has been proved to up-regulate TNF-α, IL-1 and IL-6, which contribute to the development of clinical symptoms and signs of RA[34]. Therefore, RAS inhibition could reduce inflammatory activity and various studies have proved that RAS inhibitors treatment in patients with RA are associated with improved endothelium function[17, 18, 35]. The anti-inflammation effect of RAS blockade contributes to the protection of cardiovascular risks on RA patients irrespective of lowering blood pressure[36–38].

In patients with prior MI and systolic heart failure, long-term treatment of RAS blockade had a significant 20% relative risk reduction for recurrent MI after averaging 3-year follow-up[39]. With regard to primary prevention, in patients with established cardiovascular or renal disease, around 20% risk reduction of coronary heart disease observed in placebo-controlled trials with ACEIs[40]. In our results, we found that RAS inhibitors was associated with a 30% lower risk of MI among RA patients, which was numerically higher than previous trials among non-RA patients. The different magnitude was probably explained by the very long-term observation of our study and dual anti-hypertension and anti-inflammatory properties of RAS blockade. Since individuals with RA have been reported to be at risk for coronary artery disease, early mortality and recurrent events after MI,[41, 42] our findings suggest initiation of ACEIs or ARBs for treatment of hypertension should be recommended in the current guideline[15].

We found that the risk of MI was not significantly different between control and alternative ACEIs/ARBs groups when the treatment was less than 180 days. It was probably due to intolerability or poor compliance of subjects on alternative ACEIs/ARBs in the beginning. In subgroup analyses, the risk of MI was not different with control in patients without pre-existing CVD in ACEIs or alternative ACEIs/ARB groups. Moreover, patients treated with ACEIs has similar risk of developing MI comparing with control group when they already received DMARD treatment. The underling mechanisms of these subgroup analyses are not clear but one potential explanation is the non-specific and less anti-inflammatory effect of ACEIs. Unlike ARBs, ACEIs enhances bradykinin effect by decreasing its breakdown. The elevation of bradykinin could cause an elevation of IL-1 and a reduction of IL-10, leading to additional proinflammatory effect[43, 44]. Our results, although need further confirmation, might suggest that ARBs would be preferable to ACEIs in limiting CV events in chronic inflammatory disease[45].

### Limitations

In pharmacoepidemiological studies like this, confounding by indication could lead to results with bias. The retrospective, nonrandomized nature and the imbalance in risk factors between the ACEI and ARB users and nonusers in the whole cohort also had to be taken into account. Although we employed several propensity score-based methods to balance these differences, it is certain that not all potential confounding factors were considered. For example, drug adherence and RA disease activity could not be obtained through our database. In fact, disease activity as well as the number and duration of fiares over time do contribute to the risk of CVD and we lacked data on disease activity outcomes or inflammatory status outcomes in our analysis. Finally, our primary endpoint was the time to the first occurrence of MI diagnosed via ICD9-CM coding. The accuracy of the diagnoses, which is based on the administrative data reported by physicians, may be a concern.

## Conclusions

Our analysis demonstrates a substantial protective effect of MI from the treatment with ACEIs or ARBs in patients with RA. The benefit might come from both anti-inflammatory and BP-lowering properties, supporting the recommendation of the RAS inhibitors as preferable drugs for RA patients with hypertension.

## Supporting information

S1 Table The corresponding ICD9-CM Code for comorbidities and outcomes. Abbreviation:
ICD, International Classification of Disease, Ninth Revision, Clinical Modification.
(DOCX)

S2 Table Hazard ratios (95% CI) of developing acute myocardial infarction in patients taking ACEI, ARB, or ACEI/ARB, with no RAS blockade treatment as the control group. Abbreviations
ACEIs, angiotensin converting enzyme inhibitors; ARB, angiotensin receptor blockers; CI, confidence interval; HR, hazard ratio; IPTW, inverse probability of treatment weights; MMWS, marginal mean weighting through stratification.
(DOCX)

S3 Table Hazard ratios for myocardial infarction associated with use of RAS blockade according to the follow-up period. Abbreviations
ACEIs, angiotensin converting enzyme inhibitors; ARB, angiotensin receptor blockers; CI, confidence interval; HR, hazard ratio.
(DOCX)

S4 Table Demographic and clinical characteristics of study subjects divided as user and non-user of RAS inhibitors. Abbreviations:
CAD, coronary artery disease; CCBs, calcium channel blockers, DMARD, disease modifying anti-rheumatic drugs; HTN, hypertension; NSAIDs, non-steroid anti-inflammation drugs; OADs, oral anti-diabetic drugs; PAD, peripheral artery disease; RAS, renin-angiotensin system.
(DOCX)

S5 Table The crude incidence and Hazard ratios (95% CI) of acute myocardial infarction by prescription. Abbreviations:
CI, confidence interval; HR, hazard ratio; IQR, interquartile range; PS, propensity score; RAS, renin-angiotensin system; SD, standard deviation.
(DOCX)

S1 File Raw data of our study. The data set was in the format for SPSS analysis without individual information.
(SAV)
