# Supplementary material for: Primary prevention of myocardial infarction with angiotensin-converting enzyme inhibitors and angiotensin receptor blockers in hypertensive patients with rheumatoid arthritis—A nationwide cohort study
Source: PLoS One. 2017 Dec 7;12(12):e0188720. doi: 10.1371/journal.pone.0188720 (PMC5720761; doi:10.1371/journal.pone.0188720)
Supplement: S1 Table — Abbreviation: ICD, International Classification of Disease, Ninth Revision, Clinical Modification. (DOCX) [file pone.0188720.s001.docx]

| **Disease** | **ICD9-CM code** |
| --- | --- |
| Hypertension | 401 – 405 |
| Diabetes | 249.0 – 249.91  250.0 – 250.93 |
| Dyslipidemia | 272.0 – 272.9 |
| Ischemic stroke | 434.0 – 434.91 |
| Hemorrhagic stroke | 430 – 432 |
| Coronary artery disease | 411.0 - 414.9  V17.3  V81.0 |
| Heart failure hospitalization | 428.0 – 428.3  429.9 |
| Peripheral artery disease | 250.7  443.0 – 443.9  444.2 |
| ST-segment elevation myocardial infarction | 410.0 - 410.6  410.8 |
| Non ST-segment elevation myocardial infarction | 410.7  410.9 |

**Table 1. The corresponding ICD9-CM Code for comorbidities and outcomes.**
