# Supplementary material for: Primary prevention of myocardial infarction with angiotensin-converting enzyme inhibitors and angiotensin receptor blockers in hypertensive patients with rheumatoid arthritis—A nationwide cohort study
Source: PLoS One. 2017 Dec 7;12(12):e0188720. doi: 10.1371/journal.pone.0188720 (PMC5720761; doi:10.1371/journal.pone.0188720)
Supplement: S2 Table — Abbreviations ACEIs, angiotensin converting enzyme inhibitors; ARB, angiotensin receptor blockers; CI, confidence interval; HR, hazard ratio; IPTW, inverse probability of treatment weights; MMWS, marginal mean weighting through stratification. (DOCX) [file pone.0188720.s002.docx]

**Table 2. Hazard ratios (95% CI) of developing myocardial infarction in patients taking ACEI, ARB, or ACEI/ARB, with no RAS blockade treatment as the control group**

|  | **ACEIs versus Control** | **ARBs versus Control** | **ACEIs/ARBs versus Control** |
| --- | --- | --- | --- |
| **Overall, HR (95% CI)** |  |  |  |
| Adjusted HR- IPTW | 0.815 (0.794-0.891) | 0.681 (0.604-0.702) | 0.701 (0.684-0.712) |
| Adjusted HR - MMWS | 0.821 (0.762-0.889) | 0.667 (0.593-0.711) | 0.710 (0.621-0.740) |
| *Period of treatment < 180 days* |  |  |  |
| Adjusted HR- IPTW | 0.915 (0.879-0.952) | 0.703 (0.679-0.727) | 0.904 (0.883-1.026) |
| Adjusted HR - MMWS | 0.827 (0.787-0.868) | 0.715 (0.689-0.735) | 1.132 (0.926–1.238) |
| *Period of treatment 180-360 days* |  |  |  |
| Adjusted HR- IPTW | 0.719 (0.683-0.758) | 0.750 (0.715-0.786) | 0.745 (0.719-0.794) |
| Adjusted HR - MMWS | 0.887 (0.867-0.909) | 0.852 (0.818-0.887) | 0.807 (0.740-0.974) |
| *Period of treatment > 360 days* |  |  |  |
| Adjusted HR- IPTW | 0.501 (0.484-0.519) | 0.448 (0.434-0.463) | 0.556 (0.542-0.570) |
| Adjusted HR - MMWS | 0.655 (0.637-0.675) | 0.542 (0.529-0.556) | 0.584 (0.574-0.593) |
