# Supplementary material for: Primary prevention of myocardial infarction with angiotensin-converting enzyme inhibitors and angiotensin receptor blockers in hypertensive patients with rheumatoid arthritis—A nationwide cohort study
Source: PLoS One. 2017 Dec 7;12(12):e0188720. doi: 10.1371/journal.pone.0188720 (PMC5720761; doi:10.1371/journal.pone.0188720)
Supplement: S3 Table — Abbreviations ACEIs, angiotensin converting enzyme inhibitors; ARB, angiotensin receptor blockers; CI, confidence interval; HR, hazard ratio. (DOCX) [file pone.0188720.s003.docx]

**Table 3. Hazard ratios for myocardial infarction associated with use of RAS blockade according to the follow-up period.**

|  | **ACEIs versus Control** | **ARBs versus Control** | **ACEIs/ARBs versus Control** |
| --- | --- | --- | --- |
| **Overall, HR (95% CI)** |  |  |  |
| 2-year follow-up | 0.795 (0.752-0.842) | 0.741 (0.701-0.783) | 0.854 (0.784 – 0.928) |
| 5-year follow-up | 0.950 (0.920-0.981) | 0.548 (0.528-0.568) | 0.783 (0.752-0.815) |
| 8-year follow-up | 0.977 (0.969-0.991) | 0.574 (0.557-0.592) | 0.787 (0.769-0.804) |
