# Supplementary material for: Primary prevention of myocardial infarction with angiotensin-converting enzyme inhibitors and angiotensin receptor blockers in hypertensive patients with rheumatoid arthritis—A nationwide cohort study
Source: PLoS One. 2017 Dec 7;12(12):e0188720. doi: 10.1371/journal.pone.0188720 (PMC5720761; doi:10.1371/journal.pone.0188720)
Supplement: S4 Table — Abbreviations: CAD, coronary artery disease; CCBs, calcium channel blockers, DMARD, disease modifying anti-rheumatic drugs; HTN, hypertension; NSAIDs, non-steroid anti-inflammation drugs; OADs, oral anti-diabetic drugs; PAD, peripheral artery disease; RAS, renin-angiotensin system. (DOCX) [file pone.0188720.s004.docx]

**Table 4. Demographic and clinical characteristics of study subjects divided as user and non-user of RAS inhibitors.**

|  | **Non-user** | **User of RAS inhibitors** | **P value** |
| --- | --- | --- | --- |
| n (%) | 12078 (44.2) | 15257 (55.8) |  |
| Age (mean) | 51 | 59.4 | 0.003 |
| Gender, female % | 78.3 | 78.1 | 0.120 |
| Diabetes, % | 10.7 | 34.1 | <0.001 |
| Dyslipidemia | 29.5 | 44.8 | <0.001 |
| Ischaemic stroke/TIA, % | 2.6 | 7.8 | 0.012 |
| Haemorrhagic stroke, % | 0.7 | 2.6 | 0.002 |
| CAD, % | 21.4 | 38.7 | <0.001 |
| PAD, % | 15.0 | 24.4 | 0.023 |
| Heart failure hospitalization, % | 10.6 | 16.8 | <0.001 |
| Medications |  |  |  |
| Anti-platelet | 19.8 | 34.2 | <0.001 |
| Beta-blockers | 38.6 | 66.5 | <0.001 |
| CCBs | 55.1 | 79.1 | <0.001 |
| Statin | 20.4 | 32.5 | <0.001 |
| DMARD | 48.7 | 62.1 | <0.001 |
| NSAIDs | 71.2 | 78.8 | 0.002 |
| Steroid | 70.8 | 82.8 | 0.002 |
| OADs | 10.4 | 27 | 0.001 |
| Insulin | 2.5 | 8.4 | 0.011 |
