# Supplementary material for: Primary prevention of myocardial infarction with angiotensin-converting enzyme inhibitors and angiotensin receptor blockers in hypertensive patients with rheumatoid arthritis—A nationwide cohort study
Source: PLoS One. 2017 Dec 7;12(12):e0188720. doi: 10.1371/journal.pone.0188720 (PMC5720761; doi:10.1371/journal.pone.0188720)
Supplement: S5 Table — Abbreviations: CI, confidence interval; HR, hazard ratio; IQR, interquartile range; PS, propensity score; RAS, renin-angiotensin system; SD, standard deviation. (DOCX) [file pone.0188720.s005.docx]

**Table 5. The crude incidence and Hazard ratios (95% CI) of myocardial infarction by prescription**

|  | **Non-user** | **User of RAS inhibitors** |
| --- | --- | --- |
| **Crude incidence** |  |  |
| Duration of follow-up  Median (IQR), days | 2554 (1175,4439) | 3438 (1820,5085) |
| Mean (SD), days | 2840 (2035) | 3513 (1999) |
| Incident cases - n (%) | 748 (6.2) | 892 (5.8) |
| Incidence per 1000 patient-years | 7.6 | 5.7 |
| **Overall, HR (95% CI)** |  |  |
| Adjusted HR | 1 | 0.647 (0.510 – 0.735) |
| Adjusted HR-PS adjustment | 1 | 0.657 (0.578 - 0.747) |
